# World Congress on Brain, Behavior and Emotions: São Paulo, São Paulo, Brazil June 26-29, 2013

**Published:** 2013

## THE IMPACT OF HEALTHY PARENTING AS A PROTECTIVE FACTOR FOR POSTTRAUMATIC STRESS DISORDER DEVELOPMENT IN ADULTHOOD

ADRIANO RESENDE LIMA, MARCELO FEIJÓ MELLO, SÉRGIO BAXTER ANDREOLI, VICTOR FOSSALUZA, CÉLIA MARIA DE ARAÚJO, ANDREA PAROLIN JACKOWSKI, RODRIGO AFFONSECA BRESSAN, JAIR DE JESUS MARI

UNIVERSIDADE FEDERAL DE SÃO PAULO - UNIFESP

*Introduction:* There has been a progressive recognition towards the relevance of parental bonding as a factor of risk or resiliency in neurodevelopment. Throughout the process of neurodevelopment, the corpus callosum (CC) presents relevant functions, not only for the interhemispheric connection, but also for the entire processing of emotional and mnemic stimulations - stimulations which are intimately related with the parental bonding functions. Some studies have reported reduced posterior CC area and/or integrity related to parental negligence and childhood maltreatment. A recent study has proposed alleged biological mechanisms involved in the association between precocious environmental stress and the posterior development of PTSD - the main proposed closure of which has been the verification of a continuum state, mediated by environmental mechanisms of epigenetic modulation, in between these episodes of precocious stress in childhood and the development of PTSD in adulthood. In accordance with the same study, episodes of precocious stress in life, by means of epigenetic mechanisms of sensitization of the glucocorticoid receptors, would predispose to chronic neuro-biological episodes of sensitization of adaptation to stress, with a subsequent altered standard of response of the HPA axis - a standard which is characteristic of individuals who suffer from PTSD. When exposed to traumatic situations, some people develop posttraumatic stress disorder (PTSD) while others do not manifest the disorder. In face of these findings, which inter-relate parental bonding, neuroendocrine response to stress, and brain development, in this study, we evaluated the association between parental bonding, hypothalamic-pituitary-adrenal (HPA) axis response, and callosal structural integrity, in victims of urban violence, with and without PTSD.

*Objectives:* The main aim of this study is to investigate the possible impact of healthy parenting as a protective factor for posttraumatic stress disorder development in adulthood.

*Methods:* This is a case-control study, comparing individuals who have been exposed to severe urban violent situations, and who were classified as having or not PTSD after Structured Clinical Interviews by the DSM-IV Axis I (SCID-I) interview. Parental Bonding Instrument (PBI) was applied to assess individuals’ perception of their parental relation experiences. PBI is a self-report instrument, which evaluates two dimensions of parental bonding: affection (availability, care, sensibility versus coldness and rejection), and control (protection, intrusion versus encouraging of autonomy). PBI considered optimal parenting when individuals’ perception refereed as having high affection and low control. We compared both groups, with optimal parenting and non-optimal parenting, regarding the presence of a PTSD diagnosis. In order to compare the possible differences between the groups, biological parameters were collected. Neuroendocrine response (by means of the prednisolone suppression test of the hypothalamic-pituitary-adrenal (HPA) axis) and the possible neuro-anatomical alterations (by means of the volumetry of the callous body) have been evaluated.

*Results:* Perceptions of not having a controlling mother (OR 4.84; 95%CI [2.26-10.3]; p=0.01), and of having an affective father (OR 2,46; 95%CI [1.18-5.12]; p=0.02) were protective factors for PTSD development. When we considered both parents together, perceptions of not having controlling parents was a protective factor for PTSD development (OR 2.70; 95%CI [1.10-6.63]; p=0.04). Patients with PTSD had smaller total CC area, but these differences were not statistically significant (e-value=0.34). The prednisolone suppression test showed a blunted response on PTSD+ group, with low concentrations on salivary cortisol (p=0.03), in the wake up time and thirty minutes after wake-up. Conclusion: The results of the present study point out to the impact of the healthy parental bonding, characterized by low control and high affection, and their association with hypothalamic-pituitary-adrenal (HPA) axis response, as a possible factor of protection against the development of PTSD in adulthood. The understanding of the risk factors to the psychiatric morbidity in adulthood passes by the investigation of the role play of the parental bonding in childhood, as well as by the understanding of the interfaces between the early environment and the neurodevelopment.

## PREVALENCE OF COGNITIVE IMPAIRMENT IN AFRICAN BRAZILIAN ELDERLY LIVING IN THE OLDEST ISOLATED COMMUNITY OF DESCENDENT OF SLAVES IN CENTRAL BRAZIL

DANIELLY BANDEIRA LOPES, LEONARDO CAIXETA

UNIVERSIDADE FEDERAL DE GOIÁS

*Background:* Brazil has one of the richest cultural and ethnic diversities in the world. Paradoxically, relatively little is known about cognitive functioning in aging African Brazilian and there are no studies with isolated communities of descendent of slaves (“quilombos”). The elderly population of ethnic minorities, like Afro-Brazilians, are underrepresented in dementia evaluation and care, especially because no account of cultural differences are done in most studies. The differences between ethnic groups in rates of dementia has important implications, as in health care policies and services. The study of isolated communities of ethnic minorities that conserve an ancestral way of life is an interesting model to study nature-nurture assumptions in dementia etiology.

*Objectives:* We aim to estimate the prevalence of cognitive and functional impairment in elderly living in an isolated rural community of descendent of slaves, known as ‘Kalunga’, in Central Brazil. We aim also to identify limitations in the geriatric care of minority ethnic group of descendent of slaves.

*Methods:* A crosssectional study with noninvasive methods, based on primary data of cognitive and functional elderly aged over 60 years, living in an isolated rural community of descendent of slaves, known as ‘Kalunga’, in Central Brazil. Kalunga is the oldest ‘quilombo’ in Brazil and is considered by federal law as cultural heritage site of historical value. We collected and analyzed data identification (age, gender), socio-demographic (education, place), cultural and previous morbidities (hypertension, diabetes) of participants through a semi-structured questionnaire. Data on cognitive and functional assessment were obtained through the application of the Mini-Mental State Examination (MMSE), Brief Neuropsychological Battery and the Pfeffer’s Questionnaire of Activities of Daily Living (PQADL). Those elderly with probable cognitive deficits were investigated about current geriatric care in their community. Results A total of 65 seniors were evaluated. Most subjects were male (52.3%), married (58.5%), mean age 72 years and four (6.2%) were literate. Hypertension was the prevalent comorbidity (61.5%), followed by dyslipidemia (6.2%) and diabetes mellitus (4.6%). The personal history of stroke and myocardial infarction was, respectively, 3.1% and 1.5%. Smoking and alcohol consumption accounted for 52.3% and 21.5% of respondents, respectively. The prevalence of individuals with cognitive and functional impairment was 9.2% (n=6), and the functional impairment (18.5%) was more prevalent than cognitive (12.3%). Mean MMSE and PQADL were respectively 18.83 and 4.03 points. Among these, eight individual (12.3%) had cognitive impairment according to MMSE cut off point proposed for illiterate individuals. In this sample, women had mean values of MMSE lower and PQAVD higher compared to men (CI=95%). Not a single case with cognitive disorder was accompanied by geriatric care.

*Conclusions:* Prevalence of cognitive disorder in this isolated African Brazilian community of descendent of slaves was lower than that observed in other Brazilian epidemiological studies with general population. Cultural aspects related to natural food, active exercises even among elderly and a rich cultural social life may explain the low prevalence of dementia and therefore account as a social-cultural factor intervenient in dementia etiology. This community is still invisible to any geriatric health public system, deserving more attention by government assistance.

## MUSIC EDUCATION, MUSICAL PERCEPTION SKILLS, AND THEIR RELATION TO READING ABILITY AMONG CHILDREN WITH READING DIFFICULTIES: A SYSTEMATIC REVIEW, A PRAGMATIC CLUSTER-RANDOMIZED CLINICAL TRIAL AND STRUCTURAL EQUATION MODELING

HUGO COGO-MOREIRA, CLARA REGINA BRANDÃO DE ÁVILA, GEORGE PLOUBIDIS, JAIR DE JESUS MARI

UNIVERSIDADE FEDERAL DE SÃO PAULO - UNIFESP

*Background:* Growing evidence from a range of research disciplines suggests that musical experience can have a positive effect on language and literacy abilities.

*Aims:* This thesis is constituted by three main sections: 1) a systematic review evaluating the effectiveness of music education on reading skills (oral reading skills, reading comprehension, reading fluency, phonological awareness, and spelling) in children and adolescents with dyslexia; 2) a pragmatic cluster-randomized clinical trial (RCT) to evaluate the effectiveness of music education in the improvement of reading skills and academic achievement among children (8 to 10 years of age) with reading difficulties; 3) development of an integrative model to investigate how specific domains of musical perception (temporal and melodic domains) might act as predictors of word-level reading skills among the RCT sample, which had reading difficulties, normal quotient of intelligence, and no previous exposure to music education classes.

*Methods:* Regarding the systematic review, we searched different electronic databases in June 2012 (e.g., Pubmed, Medline, ERIC, Arts and Humanities Citation Index) the WHO International Clinical Trials Registry Platform (IC-TRP), and reference lists of studies that did not employ language limitations. The intent was to include randomized controlled trials that included at least one of our primary outcomes related to the reading domain (e.g., oral reading skills, and reading and spelling measured through validated instruments). The secondary outcomes were selfesteem and academic achievement. Two authors independently screened all titles and abstracts identified through the search strategy to determine their eligibility. Regarding the RCT, 235 children with reading difficulties from 10 schools participated in a five-month, cluster randomized clinical trial (RCT) in an impoverished zone within the city of São Paulo. Reading skills and academic achievement were assessed during the school year, and five schools were chosen at random to incorporate music classes (n=114), and five served as controls (n=121). Two different methods of analysis were used to evaluate the effectiveness of the intervention: the standard method was intention-to-treat (ITT), and the other was the Complier Average Causal Effect (CACE) estimation method, which took compliance status into account. Lastly, to test the integrative model, a general-specific solution of the Montreal Battery of Evaluation of Amusia (MBEA) was regressed on word-level reading skills (rate of correct isolated words/non-words read per minute); MBEA general-specific solution is constituted by three factors: the general, temporal, and the melodic domains.

*Results:* The systematic review retrieved 851 references; no randomized controlled trials testing music education for the improvement of reading skills in children with dyslexia could be included in this review. Regarding the RCT, the ITT analyses were not very promising: only one marginal effect existed for the rate of correct real words read per minute. Indeed, considering ITT, improvements were observed in the secondary outcomes (slope of Portuguese=0.21 [p<0.001] and slope of math=0.25[p<0.001]). With regard to the CACE estimation (i.e., complier children versus non-complier children), more promising effects were observed in terms of the rate of correct words read per minute [β=13.98, p<0.001] and phonological awareness [β=19.72, p<0.001], as well as secondary outcomes (academic achievement in Portuguese [β=0.77, p<0.0001] and math [β=0.49, p<0.001] throughout the school year). The results of the integrative model were that the general and the melodic domains showed to be predictors of word-level reading.

*Conclusion:* Although music education is popular and considered a beneficial intervention, there was no evidence from randomized controlled trials to demonstrate potential advantages (or even disadvantages) of music education for improving reading skills and academic achievement until June 2012. However, although the results of the first RTC in the literature may be seen as promising, they are not, in themselves, enough for justify music lessons as public policy. Lastly, findings from the integrative model indicate that a) musical perception has two specific domains, b) the melodic domain, as a predictor of word-level reading skills among children with reading difficulties, is not an exclusive phenomenon among children with dyslexia.

## RESUMOS

### ESTUDO DA FISIOPATOLOGIA DAS ALTERAÇÕES CEREBRAIS CAUSADAS PELA SEPSE: EVIDÊNCIAS A PARTIR DE MODELO ANIMAL E DE UMA AMOSTRA DE PACIENTES

CLARISSA M. COMIM, JOÃO QUEVEDO, FELIPE DALPIZZOL

UNIVERSIDADE DO SUL DE SANTA CATARINA

*Introdução:* A sepse é uma síndrome clínica complexa, cuja causa está principalmente relacionada à resposta imune do organismo frente a um agente infeccioso. Durante seu curso, pode afetar diferentes órgãos e sistemas, inclusive o sistema nervoso central. A sepse juntamente com suas complicações está entre as principais causas de mortalidade em unidades de terapia intensiva, além de gerar sequelas permanentes aos sobreviventes..

*Objetivos:* Compreender a fisiopatologia das alterações cerebrais causadas pela sepse a partir de um modelo animal e de uma amostra de pacientes.

*Métodos:* Para alcançar os objetivos, foram feitos 4 estudos utilizando um modelo animal de sepse e 2 estudos realizados em uma amostra de pacientes admitidos na Unidade de Terapia Intensiva do Hospital São José. Os mate-riais e métodos podem ser vistos nas sessões específicas de cada artigo publicado.

*Resultados e conclusões:* Em modelo animal de sepse por CLP, houve um envolvimento cognitivo 10 dias após a indução que podem estar relacionados ao dano cognitivo e alterações metabólicas em tecido cerebral e uma diminuição dos níveis de citocinas em liquor. Além disso, em tempos mais precoces, foi demonstrado alterações do tráfego de leucócitos e da barreira hemotoencefálica, aumento dos níveis de citocinas e quimiocinas em tecido cerebral e um aumento das células apoptóticas ativadas por caspase 3 no giro denteado do hipocampo. Em uma amostra de pacientes, admitidos na UTI do Hospital São José, foi demonstrado os níveis da proteína HSP70 parecem ser modulados pelo estado oxidante do paciente com sepse e que este estado pode estar relacionado com uma maior mortalidade. Além disso, também foi demonstrado que pacientes diagnosticados com sepse que apresentaram delirium apresentaram altos níveis de TNF-α. Em conclusão, pode-se observar, a partir desses estudos preliminares, que em modelo anima, o envolvimento do sistema nervoso central parece ser mais evidenciado do que em pacientes com sepse que desenvolveram *delirium*. Novos biomarcadores devem ser estudados e correlacionados com o desfecho clínico para melhorar a compreensão desse processo.

### HEMODINÂMICA CEREBRAL AVALIADA PELA TOMOGRAFIA COMPUTADORIZADA COM ESTUDO DE PERFUSÃO EM DOEN-TES COM ACIDENTE VASCULAR ENCEFÁLICO ISQUÊMICO SUBMETIDOS À HEMICRANIECTOMIA DESCOMPRESSIVA

ROBSON LUIS OLIVEIRA DE AMORIM, EDSON BORSENG-SHU, GABRIEL GATTAS, MANOEL JACOBSEN TEIXEIRA, WELLINGSON SILVA PAIVA, ALMIR FERREIRA DE ANDRADE

HOSPITAL DAS CLÍNICAS DA FACULDADE DE MEDICINA DA UNIVERSIDADE DE SÃO PAULO

*Introdução e objetivos:* A hemicraniectomia descompressiva (HCD) para o tratamento de acidente vascular encefálico isquêmico (AVEi) hemisférico reduz a mortalidade e melhora o prognóstico funcional em alguns doentes. Este procedimento está associado à redução da pressão intracraniana. Pouco se sabe sobre o que ocorre com a hemodinâmica cerebral após a cirurgia nesse grupo de pacientes. O objetivo deste trabalho é estudar através da TC perfusão as alterações hemodinâmicas que podem ser encontradas nos doentes com AVEi após a HCD e identificar possíveis marcadores prognósticos substitutos.

*Métodos:* Foram avaliados doentes com acidente vascular encefálico isquêmico e indicação de HCD. Os parâmetros hemodinâmicos pela TC perfusão estudados no pré-operatório e em até 24h após a cirurgia foram: tempo de trânsito médio (TTM), volume sanguíneo cerebral (VSC) e fluxo sanguíneo cerebral (FSC). O desfecho primário utilizado foi melhora ou ausência de melhora hemodinâmica. Desfechos secundários avaliados foram a escala de Rankim modificada em 6 meses, que foi dicotomizada em favorável (0-3) e desfavorável (4-6); casos fatais em 1 mês e em 6 meses.Para comparar os valores pré e pós-operatórios do TTM, FSC e VSC, no hemisfério afetado e no hemisfério contra-lateral, nós utilizamos o teste T de Student pareado bicaudado para amostras normais e o teste de Wilcoxon para amostras não normais. Para avaliar o efeito do tratamento, identificamos a relação entre os valores pós e pré-operatórios do FSC, VSC e TTM (rFSC, rVSC, rTTM). Esses índices foram correlacionados com os desfechos clínicos utilizando o teste de Pearson. Resultados: Dos 27 doentes incluídos, 18 (70,3%) eram do sexo feminino e 12 (44,4%) tinham idade superior a 55 anos. A craniectomia descompressiva levou a uma redução significativa do TTM de 8,74s, IC95% 8,2-9,3 para 8,24s, IC95% 7,6-8,8 (p=0,01) e tendência a aumento do FSC de 22,37 ml/min/100g, IC95% 20,3-24,4 para 25,26 ml/min/100g, IC95% 21,7-28,9 (p=0,06). Não houve diferença significativa entre o VSC pré e pós-operatório (2,14 ml/min vs 2,26; p=0,33). Doentes com mais de 55 anos e doentes operados com mais de 48 h do ictus não apresentaram melhora significativa de nenhum parâmetro perfusional. Idade superior a 55 anos foi o preditor independente de prognóstico desfavorável (p=0,03) e o TTM pré-operatório foi preditor hemodinâmico para mortalidade em 6 meses (8,20 *vs* 9,23, p=0,04). A área sob a curva (AUC) para os valores de TTM pré e pós-operatórios foi significativa para o desfecho evolução fatal em 6 meses (ambos demonstraram AUC=0,73, IC95% 0,53-0,92, p=0,04). Os pontos de corte de 8,31 s no TTM pré-operató-rio (sensibilidade=69,2%; especificidade=71,4%) e de 8,14 s no TTM pós-operatório (sensibiidade=69,2%; especificidade=57,1%) são os mais adequados.

*Conclusões:* Este é o primeiro estudo prospectivo a avaliar os efeitos hemodinâmicos causados pela HCD no AVEi. A HCD melhora a hemodinâmica cerebral global porém, isso não ocorre em todos os doentes. Não foi observado benefício hemodinâmico nos doentes mais idosos e nos operados com mais de 48h de evolução. Apesar do impacto da melhora da perfusão ipsilateral e contralateral ao infarto poder estar relacionado com a prevenção de lesões cerebrais isquêmcas secundárias, seu efeito na melhora do prognóstico não pode ser determinado. Apesar disso, identificamos que o TTM pré e/ou pós-operatório são potenciais candidatos a marcadores substitutos para predição de desfecho fatal em 6 meses. Consequentemente, a CTP na emergência, para avaliação de doentes com AVEi que serão submetidos a HCD, torna-se uma valiosa ferramenta para avaliação dos efeitos da cirurgia e na predição de prognóstico.

### TAREFAS DE FLUÊNCIA SEMÂNTICA, FONÊMICA E DE VERBOS EM INDIVÍDUOS COM COMPROMETIMENTO COGNITIVO LEVE

VÉRONIQUE A. GUERNET STEINER, LETICIA LESSA MANSUR

FACULDADE DE MEDICINA DA UNIVERSIDADE DE SÃO PAULO

*Introdução:* A identificação precoce de indivíduos com risco de desenvolver processos demenciais é muito importante para que eles possam beneficiar-se de ações dirigidas a aspectos cognitivos e funcionais. Provas de fluência verbal são geralmente incluídas nas avaliações para o diagnóstico precoce da doença de Alzheimer pois contribuem para detectar alterações de linguagem e funções executivas. As provas de fluência verbal mais frequentemente utilizadas são as de fluência semântica (FS) e fonêmica (FF) que solicitam, respectivamente, a emissão em um minuto do maior número de palavras que pertencem a uma determinada categoria semântica ou que começam com uma letra específica. Estudos recentes têm mostrado que, com relação à FS e FF, as provas de fluência de verbos (FVe) “coisas que as pessoas podem fazer” são tarefas mais sensíveis para detectar precocemente alterações cognitivas em indivíduos com Comprometimento Cognitivo Leve (CCL). No entanto, esses dados precisam ser replicados. Embora a maioria das pesquisas costume analisar o número total de palavras produzidas em um minuto, o estudo do desempenho segundo o modelo de distribuição temporal do comportamento (Fuster, 1997), verificando a variação do número de palavras produzidas nos diferentes quartis sucessivos de 15 segundos, pode trazer informações adicionais a respeito dos processos cognitivos subjacentes a estas provas. Este modelo preconiza que, em tarefas complexas, mais recursos executivos são necessários para completá-las à medida que o tempo passa. Como cada modalidade de fluência verbal apresenta restrições linguístico-cognitivas específicas, o estudo comparativo entre indivíduos com CCL e idosos saudáveis do desempenho total e da distribuição temporal nas diferentes modalidades pode auxiliar a detectar diferenças no acesso ao conhecimento semântico por meio das funções executivas.

*Objetivos:* O presente estudo têm como objetivo comparar e detectar diferenças de desempenho entre indivíduos idosos saudáveis e com CCL nas provas de FS, FF e FVe. Adicionalmente, visa investigar os processos cognitivos subjacentes a estas provas comparando o desempenho dos grupos segundo o modelo de organização temporal do comportamento.

*Métodos:* Foram examinados 30 indivíduos com CCL pareados por idade e nível de escolaridade com indivíduos saudáveis. O desempenho de cada grupo foi analisado por meio da análise do número total de palavras corretas produzidas em um minuto nas provas de FS (animais, itens do supermercado), de FF (FAS) e de FVe (ações) e também pela distribuição temporal em cada modalidade subdividindo o tempo de produção verbal em quatro quartís de 15 segundos.

*Resultados:* Os resultados indicam diferenças significativas entre uma amostra variada de indivíduos com CCL e controles apenas para a prova de FVe, confirmando pesquisas anteriores que mostram que esta prova permite capturar precocemente os défices cognitivos associados ao CCL. O estudo da distribuição temporal permitiu detectar diferenças significativas entre os grupos nas provas de FVe, e em menor grau nas provas de FF. Nestas provas, é provável que à medida que o tempo passa e que menos palavras ficam disponíveis na rede semântica, os recursos executivos disponíveis de indivíduos com CCL não sejam suficientes para completar a tarefa. Estes resultados coincidem com os achados da literatura de que a FF e FVe dependem predominantemente do processamento executivo e do funcionamento da alça fronto-estriatal enquanto que as provas de FS estão mais frequentemente associadas ao processamento semântico e aos lobos temporais. Diferenças associadas aos recursos executivos necessários para o processamento dos verbos em relação aos substantivos podem explicar as diferenças de desempenho entre os grupos.

*Conclusão:* A FVe permite detectar precocemente a presença de alterações cognitivas no CCL e diferencia-se das provas de FS e FF. O estudo da distribuição temporal contribui para o entendimento das alterações linguístico-cognitivas subjacentes a estas provas e mostra que o que torna a FVe mais sensível às alterações cognitivas presentes no CCL é provavelmente sua forte demanda de recursos executivos. Diferenças entre o processamento de verbos e de substantivos podem explicar a maior demanda de recursos executivos na FVe.

### EFEITO DO TREINAMENTO MUSICAL EM CAPACIDADES COGNITIVAS VISUAIS: ATENÇÃO E MEMÓRIA

*ANA CAROLINA OLIVEIRA E RODRIGUES, MAUR*Í*CIO ALVES LOUREIRO, PAULO CARAMELLI*

FACULDADE DE MEDICINA DA UNIVERSIDADE FEDERAL DE MINAS GERAIS

A influência da música sobre a função cerebral tem sido alvo da investigação de neurocientistas e músicos desde a década de 1990. Muitas pesquisas têm descrito, em músicos, alterações neuroplásticas, estruturais e funcionais, decorrentes da prática musical prolongada, envolvendo áreas como corpo caloso, hipocampo, cerebelo, cápsula interna, tronco encefálico, além de diversas regiões do córtex cerebral. Tais processos de neuroplasticidade estrutural e funcional podem influenciar o funcionamento cognitivo, produzindo também diferenças entre músicos e não-músicos. Várias pesquisas têm relatado, em crianças, associações positivas entre o estudo formal da música e capacidades cognitivas pertencentes ao domínio não-musical, como raciocínio verbal, matemático e visual-espacial, bem como inteligência geral. Embora os efeitos do treinamento musical sobre funções cognitivas tenham sido mais bem documentados em crianças, estudos envolvendo adultos também têm mostrado diferenças entre os grupos em diferentes domínios cognitivos, especialmente em relação à cognição visual. O objetivo geral do trabalho consistiu em investigar se o treinamento musical intensivo está associado a capacidades cognitivas visuais aumentadas: atenção visual, em três modalidades - seletiva, dividida e sustentada - e memória visual. Dois grupos de voluntários, equiparados em relação à idade, gênero e escolaridade, participaram do estudo: músicos (n = 38), membros permanentes de duas importantes orquestras brasileiras - Orquestra Filarmônica de Minas Gerais e Orquestra Sinfônica de Minas Gerais - e não-músicos (n = 38), profissionais e estudantes de diversas áreas do conhecimento. Inicialmente, foram administrados os seguintes formulários: questionário sociodemográfico,­ para caracterização de cada indivíduo; Escala de Sonolência Epworth, para avaliação da sonolência diurna; e parte do *Mini International Neuropsychiatric* *Interview*, para investigação de possíveis transtornos psiquiátricos. Não foram verificadas diferenças significativas entre músicos e não-músicos em relação à sonolência diurna (t(74)= -1,95; p=0,055) e horas de sono por noite (t(74)=1,06; p=0,293). Também não houve indício de Episódio Depressivo Maior e Dependência/Abuso de Álcool para nenhum indivíduo. Entretanto, foi verificado indício de Transtorno de Ansiedade Generalizada em quatro indivíduos - um músico e três não-músicos. Nenhum voluntário mencionou fazer uso de medicamento com ação sobre o sistema nervoso central. Posteriormente, os indivíduos foram submetidos a cinco testes neuropsicológicos computadorizados, elaborados com o auxílio do programa E-Prime: três testes de atenção visual, sendo um para cada modalidade atencional, um teste de memória visual e um teste de tempo de reação simples, os quais avaliaram tempo de reação e acurácia. Os músicos mostraram melhor desempenho em quatro variáveis dos testes de atenção visual: acurácia no teste de atenção seletiva (t(74)=2,00; p=0,049), tempo de reação na tarefa 1 e número de erros na tarefa 2 no teste de atenção dividida (t(74)= -2,26; p=0,026 e U=493,50; p=0,011, respectivamente), e tempo de reação no teste de atenção sustentada (U=406,50; p=0,001). Além disso, os músicos apresentaram resultado superior em três variáveis do teste de memória visual: tempo de reação no teste como um todo (t(74)= -2,20; p=0,030), tempo de reação na parte 1 (t(74)= -2,15; p=0,035) e tempo de reação na parte 2 (t(74)= -2,01; p=0,048) do teste. O desempenho dos músicos nos testes de atenção e memória não pode ser explicado apenas por melhor integração sensório-motora, uma vez que não houve diferença entre os grupos no teste de tempo de reação simples (t(74)= -1,86; p=0,067). Além disso, foram verificadas correlações significativas entre variáveis relacionadas à experiência musical - idade de início dos estudos musicais e tempo de estudo individual com instrumento por dia - e algumas variáveis dos testes de atenção e memória visuais. Nossos resultados sugerem haver maior capacidade de atenção visual, em diferentes modalidades, em músicos. O melhor desempenho dos músicos no teste de memória visual também pode indicar maior eficiência dos processos atencionais, já que as diferenças foram observadas apenas em relação aos tempos de reação. Este estudo poderá contribuir para demonstrar possíveis benefícios cognitivos do treinamento musical prolongado, com possíveis implicações para as áreas de saúde e educação.

### ESTUDO LONGITUDINAL COMPARATIVO SOBRE OS EFEITOS NEUROCOGNITIVOS E CLÍNICOS DA PSICOTERAPIA PSICODI-NÂMICA DE LONGO PRAZO, DA TERAPIA COM FLUOXETINA E DO TRATAMENTO COMBINADO EM ADULTOS DEPRIMIDOS

ANDRE GOETTEMS BASTOS, LUCIANO SANTOS PINTO GUIMARÃES, CLARISSA MARCELI TRENTINI

UNIVERSIDADE FEDERAL DO RIO GRANDE

Estimativas da Organização Mundial de Saúde mostram que no ano de 2020 a depressão será a segunda doença mais presente em todas as faixas etárias, em ambos os sexos, em todo o mundo. Trata-se de uma doença grave, com repercussões importantes em diversas áreas, entre elas a cognitiva. As principais alterações cognitivas encontradas em pacientes deprimidos são prejuízos no processamento e organização do conteúdo perceptual, na memória de trabalho, na capacidade de atenção, nas funções executivas, no controle cognitivo, nos processos inibitórios e na velocidade de processamento cognitivo. O próprio *Diagnostic* *and Statistical Manual of Mental Disorders* (DSM-IV-TR) inclui, entre os critérios diagnósticos para depressão, retardo psicomotor e concentração diminuída. Apesar de diversos ensaios clínicos randomizados demonstrarem a eficácia clínica (diminuição dos sintomas e recuperação funcional) de diferentes tratamentos para depressão, existem poucos estudos clínicos randomizados que examinaram e compararam a eficácia clínica e também os efeitos neurocognitivos dos diferentes tratamentos. O presente trabalho teve os seguintes objetivos: Examinar e comparar a eficácia clínica e os efeitos neurocognitivos da psicoterapia psicodinâmica de longo prazo (LTPP), da terapia com fluoxetina (FLU), e da combinação de ambos (COM); Identificar variáveis sociodemográficas e/ou neurocognitivas que poderiam estar associadas à resposta clínica dos pacientes ao longo dos três tipos de tratamento; Identificar as variáveis que possuem potencial prognóstico, prescritivo e/ou moderador de desfecho clínico.

*Métodos:* 272 pacientes deprimidos com intensidade moderada de sintomas foram randomizados para receber LTPP, FLU ou COM, por um período de 24 meses. O Inventário de Depressão de Beck (BDI) foi usado para monitorar a evolução e o desfecho clínico, e a terceira edição da Escala Wechsler de Inteligência (WAIS-III) foi a bateria de testes neuropsicológicos utilizada para o monitoramento neuro-cognitivo dos pacientes. Os pacientes foram avaliados em cinco momentos (pré-tratamento, seis, 12, 18 e 24 meses de tratamento). Todos os tratamentos tiveram 24 meses de duração. As análises estatísticas foram executadas através de modelos mistos, seguindo a lógica intention-to-treat, e descrevendo e comparando aritmeticamente a relação entre os resultados do BDI, da WAIS-III e o tempo, bem como sua interação dentro dos grupos de tratamento e entre eles.

*Resultados:* Os resultados indicaram que todos os tratamentos foram eficazes na redução dos escores do BDI dos pacientes. No entanto, pacientes dos grupos LTPP e COM mostraram resultados clínicos mais acentuados do que os pacientes recebendo FLU. Também foram encontradas alterações neurocognitivas nos pacientes ao longo dos tratamentos, com diferenças estatisticamente significativas. Pacientes dos grupos LTPP e COM tiveram alterações neurocognitivas positivas em funções cognitivas específicas, na comparação com os pacientes de FLU. Além disso, foram identificadas seis variáveis preditivas de desfecho de tratamento: três variáveis prognósticas relacionadas à memória de trabalho e ao raciocínio abstrato; uma variável prescritiva relacionada à memória de trabalho; e duas variáveis moderadoras, o raciocínio abstrato e a velocidade de processamento cognitivo.

*Conclusões:* LTPP e COM são mais eficazes do que FLU na redução dos sintomas clínicos e na melhora neurocognitiva de pacientes com depressão moderada. O resultado da comparação entre LTPP e COM depende do critério utilizado para apontar o desfecho (tamanho de efeito, percentual de melhora, número de pacientes abaixo do ponto de corte para desfecho positivo, ou NNT). Variáveis neurocognitivas servem como preditivas de desfecho: O nível de funcionamento da memória de trabalho identificou subgrupos que parecem responder melhor à LTPP e COM do que à FLU, sendo considerado prescritivo; O raciocínio abstrato e velocidade de processamento foram considerados moderadores de desfecho, pois influenciaram na magnitude e/ou na direção da melhora clínica; A memória de trabalho e o raciocínio abstrato foram prognósticos, pois identificaram subgrupos que poderiam se beneficiar de estratégias terapêuticas específicas. Novos estudos são sugeridos.

### FUNÇÕES NEUROPSICOLÓGICAS EXECUTIVAS PÓS ACIDENTE VASCULAR ENCEFÁLICO HEMORRÁGICO

ANA PAULA AFONSO CAMARGO, CARMEN MARIA BUENO NEME, MARIA DE LOURDES MERIGHI TABAQUIM

UNIVERSIDADE ESTADUAL PAULISTA (UNESP) E FACULDADE MÉTODO DE SÃO PAULO (FAMESP)

Os acidentes vasculares encefálicos constituem-se um sério problema de saúde, e dados sobre sua prevalência mundial têm chamado à atenção. Dentre os subtipos de AVE, a hemorragia intraparenquimatosa (HIP) configura-se a de pior prognóstico. Frequentes déficits cognitivos e a significativa incidência de sintomatologia depressiva têm sido associados a este insulto neurológico. Entretanto, os expressivos índices de morbimortalidade implicam em dificuldades para a execução de estudos inerentes ao funcionamento neuropsicológico, pós lesão. Este estudo teve como objetivo principal investigar as funções neuropsicológicas executivas, em sujeitos pós acidente vascular encefálico hemorrágico. Como objetivo secundário, buscou verificar as relações entre sintomas depressivos pós infarto cerebral hemorrágico intraparenquimatoso, respostas cognitivas e topografia lesional. Para isso, foram triados 123 pacientes admitidos de modo consecutivo, pelo Serviço de Neurocirurgia do Hospital de Base de Bauru. Destes, foram elegíveis 12 sujeitos, com HIP espontânea e primária, de ambos os sexos, com idade média de 58,5 anos. A avaliação neuropsicológica das funções executivas consistiu na aplicação do Wisconsin Card Sorting Test; do Subteste Dígitos (WAIS); Teste Blocos de Corsi; Trail Making Test; Stroop Test; e das provas de fluência verbal fonêmica e categoria semântica. O grau de gravidade do AVE foi medido pela Escala de AVE do National Institutes of Health, Escala de Coma de Glasgow e pelo Escore de AVCH. O comprometimento nas AVDs foi medido pelo Índice Barthel, e a sintomatologia depressiva pelo Inventário de Depressão de Beck. As avaliações ocorreram em dois momentos: a primeira, em média 50,4 horas, em internação hospitalar, e a segunda em média 36 dias, após o AVE. Os exames de neuroimagem, por Tomografia Computadorizada de Crânio, foram realizados em admissão hospitalar, dada a hipótese diagnóstica de AVE. Os dados foram analisados pelo *Statistical Package for Social Sciences*, e a correlação de Spearman foi calculada para a verificação do grau de relacionamento entre as variáveis, considerando um nível de significância de 0,05. Os resultados demonstraram que 91,6% da população estudada apresentou comprometimento das funções executivas, 75% em atividades de vida diárias, e em 66,6% dos sujeitos foi identificada a sintomatologia depressiva, na fase aguda pós HIP. Verificou-se maior incidência de lesão em regiões profundas (66,7%), quando comparadas às lesões em áreas lobares (33,3%). Embora relações estatisticamente significantes tenham sido encontradas apenas entre lesões profundas e processos atencionais deficitários, bem como entre sintomas depressivos e fluência verbal, com p<0,05. A incidência de Hemorragia Intraparenquimatosa associou-se a disfunção executiva, considerando que hemorragias em sítios profundos apresentaram-se relacionadas aos desempenhos mais rebaixados de funcionamento executivo. Sujeitos pós AVE mostraram-se vulneráveis à sintomatologia depressiva, nos casos com topografia profunda de lesão, com maior frequência de relações entre os sintomas depressivos e prejuízos nas funções executivas.

### RELEVÂNCIA DE VARIANTES GENÉTICAS PARA ENOS, ELN, COL1A2, ENG E VEGF EM ANEURISMA INTRACRANIANO FAMILIAL

MICHELE LIMA GREGÓRIO, WALDIR ANTONIO TOGNOLA

FACULDADE DE MEDICINA DE SÃO JOSÉ DO RIO PRETO - FAMERP

*Introdução:* Fatores de risco ambientais como tabagismo, etilismo, além de fatores genéticos envolvidos no processo de formação da parede do vaso arterial têm influência na formação de aneurismas intracranianos (AI).

*Objetivos:* Avaliar a associação de polimorfismos genéticos para óxido nítrico sintase endotelial (eNOS-G894T), colágeno (COL1A2-Ala459Pro), elastina (ELN-A442G), endoglina (ENG-Ins/Del) e fator de crescimento endotelial vascular (VEGF-C936T) com AI familial e esporádico e seus respectivos familiares em primeiro grau; avaliar a razão de chance para aneurisma intracraniano em portadores dos polimorfismos estudados; analisar a relação entre os polimorfismos estudados, hábitos de vida, presença de hipertensão arterial sistêmica (HAS), diabetes melito (DM), além de características morfológicas dos AI destes indivíduos.

*Metodologia:* Foram estudados 847 indivíduos, assim distribuídos: G1 - 43 pacientes com AI familial; G2 - 177 familiares em primeiro grau de G1; G3 - 115 pacientes com diagnóstico de AI esporádico; G4 - 276 familiares em primeiro grau de G3; G5 - 106 indivíduos sem a doença; G6 - 130 familiares em primeiro grau de G5. A análise dos referidos polimorfismos foi realizada por PCR/RFLP. Admitiu-se nível de significância P 0,05). Tabagismo/Etilismo x genótipos: Em G3 houve maior freqüência da combinação do genótipo de risco (_/C do COL1A2) + tabagistas comparado a G5 (P=0,014), enquanto para ENG-Ins/Del a combinação do genótipo selvagem (Wt/Wt - proteção) + não tabagismo prevaleceu em G5 comparado a G1 (P=0,008). Em G1 e G3 a combinação genótipo de risco _/T (VEGF-C936T) + tabagismo foi mais freqüente comparado a G5 (P0,05).

*Conclusão:* Polimorfismos envolvidos na gênese da vasculatura arterial cerebral, incluindo eNOS-G894T, ELN -A442G, ENG-Ins/Del, COL1A2-Ala459Pro e VEGF-C936T influenciam na manutenção da integridade vascular, tendo em vista sua associação em famílias com AI familial ou esporádico. Variantes mutantes de eNOS-G894T e ENG-Ins/ Del podem influenciar no AI, enquanto VEGF representado pelo homozigoto selvagem C/C parece exercer efeito protetor. Os polimorfismos de COL1A2-Ala459Pro e ENG-Ins/ Del parecem prevalecer nos familiares de pacientes com aneurisma familial, diferenciando familiares de pacientes com AI familial e controles, confirmando a contribuição do estudo de famílias na avaliação de fatores de risco para AI familial ou esporádica.

### IMPORTÂNCIA DA AVALIAÇÃO NEUROPSICOLÓGICA NO DIAGNÓSTICO DA DOENCA DE ALZHEIMER: UM ESTUDO NUMA POPULAÇÃO DE PACIENTES DIAGNOSTICADOS COMO PROVÁVEL DOENÇA DE ALZHEIMER EM PALMAS - TO

ANALUCY A.V. DE OLIVEIRA

UNIVERSIDADE DE BRASÍLIA - UNB

A análise dos déficits cognitivos para detectação de comprometimentos cognitivos é considerada de grande interesse devido às repercussões que elas podem ter no diagnóstico prematuro de demências e mais especificamente na Doença de Alzheimer (DA). No entanto, pouco é sabido sobre as dificuldades em realizar um diagnóstico diferencial correto e precoce em certas regiões do país, especificamente em Palmas-TO, região Norte. que as características do envelhecimento normal, havendo menor informação sobre essa associação do declínio cognitivo com o Comprometimento Cognitivo Leve (CCL), e em particular, na doença de Alzheimer (DA). Dessa forma, o objetivo principal desse estudo foi avaliar o comprometimento cognitivo de uma amostra de pacientes com DA, a partir da utilização de testes neuropsicológicos tradicionais. Especificamente, foi investigado se as condições demográficas e o índice intelectual destes pacientes influencia os resultados obtidos em sua avaliação em pacientes diagnosticados com prováveis DA. Foram selecionados 100 sujeitos entre 60 e 93 anos de idade, sendo 36 idosos hígidos voluntários da comunidade e 70 pacientes com suspeita de DA leve-moderado, segundo os critérios do NINCDS-ADRDA e do *Clinical Dementia* *Rating* - CDR da comunidade local, pacientes encaminhados por médicos neurologistas, psiquiatras e geriatras. Inicialmente, os dois grupos foram avaliados mediante uma bateria neuropsicológica para obter maior compreensão do funcionamento cognitivo de cada um dos participantes e como critério de seleção da amostra. Posteriormente, foram administrados os testes pertencentes a avaliação neuropsicológica: MEEM, Escala Mattis, IQCODE, FAZ de Fluência Verbal, Bateria de Boston, e do Relógio. De forma geral, os resultados encontrados referentes à acurácia indicaram que os pacientes diagnosticados como provável DA exibiram desempenho superior em muitos itens da avaliação na bateria neuropsicológica. Considerando as condições de Intelecto e sociográficas. No entanto, terem sido diagnosticados previamente como DA.Esses resultados sugerem que os pacientes na realidade estariam melhor caracterizados como CCL ou DA incipiente. A implementação de um diagnóstico mais preciso na fase pré-clínica poderia trazer inúmeros benefícios quanto a terapêutica dos casos de CCL no sentido de evitar ou retardar o avanço do declínio cognitivo para DA. *Palavras chave:* doença de Alzheimer, declínio cognitivo, memória operacional.

### IMPLICAÇÕES DA POSTURA DISTÔNICA NA EPILEPSIA DO LOBO TEMPORAL COM ESCLEROSE MESIAL TEMPORAL

CARINA GONÇALVES PEDROSO UCHIDA

UNIVERSIDADE FEDERAL DE SÃO PAULO

*Introdução:* Epilepsia do lobo temporal (ELT) com esclerose mesial temporal (ELT-EMT) cursando com crises farmacorresistentes é uma síndrome na qual os resultados do tratamento cirúrgico são superiores aos do tratamento clínico, com bom controle de crises em 60-70% dos casos. Porém, esta porcentagem diminui com longos períodos de seguimento pós-operatório. Postura distônica ictal unilateral (PD) é o sinal lateralizatório mais confiável na ELT, e sua ocorrência varia entre 15 e 87% dos pacientes, sendo contralateral ao lobo epileptogênico em mais de 90% dos casos. Embora a ocorrência de PD durante a monitorização pré-operatória por vídeo-EEG seja importante para a correta lateralização do hemisfério epileptogênico na ELT-EMT, seu impacto no prognóstico cirúrgico quanto ao controle de crises permanece controverso. Ainda, PD foi recentemente associada a um sistema homoestático cerebral endógeno que evita a evolução da crise parcial complexa (CPC) para crise tônico-clônica generalizada (TCG).

*Objetivo:* Analisar o impacto da PD no prognóstico pós-cirúrgico de pacientes com ELT-EMT, e verificar sua influência na evolução de crises parciais para crises TCG durante o vídeo-EEG pré-operatório destes pacientes.

*Métodos:* Todas as CPC disponíveis e em boas condições de análise dos pacientes submetidos à lobectomia temporal anterior (LTA) na UNIFESP entre 2002 e 2010, com pelo menos um ano de seguimento pós-cirúrgico, foram retrospectivamente analisadas e classificadas como tendo ou não PD, e evoluindo ou não para crises TCG. Os correlatos semiológicos da PD, o intervalo de tempo transcorrido entre a crise analisada e a crise anterior e o esquema de drogas antiepilépticas (DAEs) prescrito antes de cada crise foram avaliados. Auras isoladas foram excluídas da análise. O prognóstico cirúrgico quanto ao controle de crises foi aferido de acordo com a escala de Engel no primeiro ano após a LTA, e na última visita ambulatorial.

*Resultados:* 527 CPC de 171 pacientes foram analisadas. Cinqüenta e oito dos 171 pacientes (34%) apresentaram PD, 91,5% sempre unilateral e contralateral ao lado operado. PD foi associada a crises mais breves (p=0,007) e diminuiu a probabilidade de evolução de CPC para crises TCG (p=0,001), mesmo durante a retirada de DAEs (p=0,002).

*Conclusão:* Não houve associação entre PD e prognóstico cirúrgico quanto ao controle de crises, mesmo em longos períodos de seguimento pós-operatório. Nossos dados suportam a hipótese de que a ocorrência da PD poderia ser um marcador clínico da ativação de redes neuronais específicas que influenciam no controle das crises, evitando a evolução de CPC para crises TCG, inclusive quando da retirada das DAEs. *Palavras-chave:* epilepsia do lobo temporal, distonia, prognóstico, crises convulsivas generalizadas, video-EEG.

### SERÁ POSSÍVEL IDENTIFICAR INDIVÍDUOS COM DEMÊNCIA PELA FRASE DO MINI-MENTAL?

WELMA WILDES AMORIM, CRISTIANE NAMIUTI TEMPONI, NIRVANA FERRAZ SANTOS SAMPAIO, IGOR ALOISIO GARCEZ ZAMILUTE, DÉBORAH CARVALHO CAVALCANTE, VANESSA LIMA LINS

UNIVERSIDADE ESTADUAL DO SUDOESTE DA BAHIA

Dentre as questões do Mini Exame do Estado Mental (MEEM) é solicitado ao paciente para escrever uma frase. Na prática, observamos dificuldades na escrita da frase que não significam necessariamente déficit cognitivo. Dessa forma, o objetivo desse trabalho é comparar o erro da frase de idosos com demência e sem demência.

*Métodos:* Os erros das frases do MEEM escrita por idosos sem demência do “Estudo de Avaliação Multidimensional de Idosos Atendidos em Unidades de Saúde da Família (USF)” de um município do nordeste brasileiro foram comparados com os erros das frases do MEEM de idosos com demência e com escolaridade maior ou igual a 1 ano atendidos no ambulatório de geriatria da Universidade Estadual do Sudoeste da Bahia (UESB). Foi realizada uma análise linguística das frases levando-se em conta erros gráficos, de segmentação, agramaticalidades (erros sintáticos e semânticos que levam ao estranhamento da construção da frase pelos falantes e que não são naturalmente gerados por falantes da língua). O conceito de gramática considerado não se refere às normas do bem falar e escrever, mas ao conjunto de instruções abstratas que faz parte do conhecimento que temos sobre a nossa língua e que nos permite juntar sons e sentidos de forma clara para expressar pensamentos.

*Resultados:* Foram analisadas as frases de 21 idosos sem demência e encontrados erros gráficos em 12 (MJS, masculino, 71 anos, 0 escolaridade: “EU GOS DE TRABALHA”; ECL, feminino, 82 anos, 4 anos de escolaridade: “HONTEM FRITEI UMAS BALÂS PARA UMAS VISITAS”; LGD, feminino, 60 anos, 4 anos de escolaridade: “VOCE E BOA”; EOS, feminino, 77 anos, 0 escolaridade: “RECIB UMA VISITA DE ESTUDANTI”; MFS, feminino, 68 anos, 2 anos de escolaridade: “EU GOSTO MUITO DE JERZUIS”; ESM, feminino, 62 anos, 03 anos de escolaridade: “GOSTEI QUE VAIS VEI AQUI”; FMO, masculino, 66 anos, 5 anos de escolaridade: “QUERO GANHA DINHIRO”; MFS, feminino, 63 anos, 2 anos de escolaridade: “EU GOSTO DE TRABLIA”; JXS, feminino, 73 anos, 0 escolaridade: “FIZ CAMIA NA LAGO ADORO FAZER CAMIADO E SINTO BEM”; CRM, feminino, 64 anos, 4 anos de escolaridade: “VOU NA ROCA VIZITA MINHAS IRMÃS”; MBF, feminino, 69 anos, 5 anos de escolaridade: “SAÚDADES DE MEU PAI” e EFS, feminino, 66 anos, 5 anos de escolaridade: “MININAS GOSTEI MUITO DAS SUAS PUZESA”. Foram analisadas as frases de 10 idosos escolarizados e com demência e encontrados erros de grafia, segmentação e agramaticalidade em 3 frases (JRS, masculino, 75 anos, 1 ano de escolaridade: “MAIS VALE 1 ANDO MAIS VAL UM NÃ MÕ DE VUNDO”; FCS, feminino, 88 anos, 4 anos de escolaridade: “MINHA SOBRIA EU NÃO SEI TABOM” e LJAB, feminino, 77 anos, 8 anos de escolaridade: “ABRE OS OLHOS NO PAPEL NA HISTÓRIA [ININT]”) e apenas erro de grafia em uma frase (EMSV, feminino, 62 anos, 11 anos de escolaridade: “GOSTEI DOS SEUS TRABALHO”).

*Discussão:* Nas frases dos idosos sem demência, ocorrem erros gráficos semelhantes aos erros de crianças em processo de aquisição da escrita. Isso se deve possivelmente ao baixo grau de escolarização desses indivíduos, podendo indicar estagnação do processo de aquisição da escrita, como “mãos inábeis”. A presença de erros gráficos e agramaticalidades nas frases dos idosos com demência podem revelar perda de conhecimento sobre o funcionamento da língua. O caso do idoso com demência cuja frase só apresentava erro gráfico destaca-se, pois, que tal erro não é compatível com seu grau de escolaridade, sugerindo um retrocesso à fase inicial de aquisição da escrita.

*Conclusão:* Tanto as frases dos idosos com e sem demência apresentaram erros gráficos, porém, só ocorreram agramaticalidades nos idosos com demência. Esse fato está em consonância com a teoria de Chomsky que afirma que os falantes nativos de uma língua, com sua competência linguística intacta, independente do seu grau de instrução, só produzem sentenças gramaticais.

### TEMPO DE RECORRÊNCIA E DIFERENÇAS ENTRE CRISES EPILÉTICAS PRECOCES OU TARDIAS APÓS CIRURGIA PARA EPILEPSIA DO LOBO TEMPORAL

EDUARDO GOELLNER, MARINO MUXFELDT BIANCHIN, ANDREW G. PARRENT, JORGE G. BURNEO, DAVID A. STEVEN

HOSPITAL MÃE DE DEUS

*Introdução:* A recorrência de crises epiléticas pós cirurgia para tratamento da epilepsia do lobo temporal tem sido classificada como precoce ou tardia dependendo do tempo da primeira crise depois do procedimento. Contudo, este tempo de recorrência é variado, sendo arbitrariamente definido nos artigos científicos.

*Objetivos:* Nós desenvolvemos um modelo matemático que pode identificar pacientes com chance de recorrência de crises pós-operatórias precoces ou tardias. Após, analisamos os dois grupos para identificar as diferenças clínicas, eletrofisiológicas e de neuroimagem entre estes pacientes.

*Métodos:* Uma coorte histórica com 247 pacientes tratados com cirurgia para epilepsia do lobo temporal foi estudada. Dentre os paciente onde as crises epiléticas retornaram, utilizamos o tempo de recorrência em uma curva ROC para avaliar com maior acurácia o melhor período para predizer o prognóstico cirúrgico a longo prazo. Com isto, dividimos os pacientes em dois grupos: os de recorrência precoce e os de recorrência tardia. Após, nós analisamos as diferenças clínicas e radiológicas entre estes pacientes.

*Resultados:* As crises epiléticas retornaram em 107 (48.9%) pacientes. A curva ROC mostrou que 6 meses era o tempo onde o prognóstico a longo prazo poderia ser determinado com maior acurácia (AUC=0.761; sensibilidade=78.8%; especificidade=72.1%). Nós observamos que nos pacientes onde a recorrência ocorreu nos primeiros 6 meses após a cirurgia, a epilepsia começou mais precocemente (OR: 6.034; CI95%: 1.056-11.013; p=0.018), estes pacientes possuíam um pior prognóstico (6.849; 2.538- 18.518; p=0.001), necessitaram de maior número de medicação antiepilética após o procedimento (2.07; 1.162- 9.345; p=0.013) e mais frequentemente foram submetidos a uma nova cirurgia para controle de crises (9.592; 1.181- 77.877; p=0.021). Nos pacientes com recorrência tardia, as crises epiléticas eram mais comumente associadas a fatores desencadeantes (9.615; 3.521-26.315; p<001).

*Conclusão:* Pacientes com recorrências de crises epiléticas precoces ou tardias possuem diferentes características que podem estar relacionadas a diferenças entre os tipos de zonas epileptogênicas, eficácia dos procedimentos cirúrgicos ou mesmo à própria epileptogenicidade. A existência de tais disparidades podem ajudar a explicar os diferentes padrões de recorrência de crises pós cirurgia para epilepsia, auxiliando no planejamento do tratamento destes pacientes a longo prazo.

### IMPORTANTE PAPEL DE VARIANTES GENÉTICAS ENVOLVIDAS NO METABOLISMO DE LIPÍDIOS NA DOENÇA DE ALZHEIMER DO TIPO TARDIO

MARCELA AUGUSTA DE SOUZA PINHEL, DOROTÉIA ROSSI SILVA SOUZA

FACULDADE DE MEDICINA DE SÃO JOSÉ DO RIO PRETO - FAMERP

*Introdução:* A doença de Alzheimer (DA) é a principal causa de demência nos países ocidentais. Além do polimorfismo genético da apolipoproteína E (apo E), há referência da participação do colesterol na regulação e clivagem da proteína precursora da β-amilóide (APP), importante para desenvolvimento de DA. Nesse caso, outros genes relacionados com o metabolismo de lipoproteínas e colesterol, além da apo E, também têm sido estudados. Incluim-se *adenosine triphosphate binding cassette transporter* 1 (ABCA1), cujo polimorfismo associa-se à alterações no efluxo do colesterol, no metabolismo de apo E e secreção de β-amilóide, e o gene para proteína de transferência do éster de colesterol (CETP), a qual media a transferência de éster de colesterol de lipoproteínas de alta densidade (HDL) para lipoproteínas ricas em triglicérides (TG), delas recebendo em troca TG.

*Objetivos:* Avaliar os polimorfismos APOE-HhaI, ABCA-1-StyI e CETP-TaqIB relacionados com perfil lipídico, em pacientes com DA do tipo tardio.

*Casuística e métodos:* Foram selecionados 322 indivíduos com mais de 65 anos distribuídos em: Grupo de Estudo (GE) - 166 pacientes com DA; Grupo Controle (GC) - 156 idosos sem demência. Foram coletadas amostras de sangue periférico para análise dos polimorfismos propostos por reação em cadeia da polimerase (PCR) e polimorfismo de tamanho dos fragmentos de restrição (RFLP), além do perfil bioquímico incluindo TG, colesterol total (CT), fração de colesterol de lipoproteína de baixa (LDLc), muito baixa (VLDLc) e alta (HDLc) densidade. A análise estatística compreendeu os testes de Fisher ou Qui Quadrado, test t ou Mann-Whitney e Kramer-Tukey. Foi admitido erro alfa de 5%, com nível de significância para P<0,05.

*Resultados:* Os genótipos APOE*_/4 (APOE-HhaI) e G/G (ABCA1-StyI) prevaleceram no GE (30% e 46%, respectivamente) comparado ao GC (8%, P<0,0001 e 37%, P=0,017; respectivamente). As distribuições alélicas e genotípicas de CETP-TaqIB foram semelhantes em ambos os grupos. Pacientes com DA mostraram prevalência da combinação do genótipo de risco para os polimorfismos de APOE-HhaI, ABCA1-StyI e CETP-TaqIB(APOE*/_4 +GG + B1B1) (23%) em relação aos controles (7%, P=0,002). GE apresentou níveis plasmáticos mais elevados de CT e LDLc (207,3±47,8 mg/dL; 20,3±46,5 mg/dL, respectivamente), quando comparado ao GC (187,2±60,2 mg/dL; 102,5±51,2 mg/dL P=0,014; P=0,018, respectivamente), principalmente nos portadores do genótipo APOE*4 (LDLc= 143,5±65,3 mg/dL) versus portadores do genótipo APOE*3/3 (111,3±34,1 mg/dL; P=0,015). Além disso, o alelo G (ABCA1-StyI) foi associado a níveis aumentados de CT (201,2±50,5 mg/dL) e LDLc (120,6±47,0 mg/dL) em pacientes com DA comparado com controles (181,0±56,1 mg/dL, P=0,019 e 97,4±46,0 mg/dL, P=0,002, respectivamente). Conclusões- Os polimorfismos APOE-HhaI e AB-CA1-StyI associam-se com a doença de Alzheimer do tipo tardio, diferenciando também os grupos com e sem DA considerando alterações no perfil lipídico plasmático, o que deve ser esclarecido também em nível de sistema nervoso central.

### AGRESSIVIDADE EM PACIENTES PSIQUIÁTRICOS INTERNADOS

VITOR CRESTANI CALEGARO, CHRISTINA CHITOLINA SCHETINGER, AMANDA BOLSON DOTTO, DENISE FREITAS, ANDERSON BARCELLOS, ANDREI GARZIERA VALERIO, LUMA CANZIAN, ANGELO BATISTA MIRALHA DA CUNHA

UNIVERSIDADE FEDERAL DE SANTA MARIA

A agressividade ocorrida durante a internação psiquiátrica gera danos físicos e psicológicos tanto para os pacientes quanto para a equipe de saúde. As pesquisas atuais não permitem a generalização dos resultados para a realidade local. Os objetivos desta pesquisa são: (1) determinar a prevalência da agressividade nas primeiras 24 horas de internação psiquiátrica, (2) relacionar fatores de risco com o comportamento agressivo e (3) testar diferenças psicopatológicas entre os grupos. Trata-se de um estudo transversal, realizado no Serviço de Psiquiatria do Hospital Universitário de Santa Maria, com os pacientes internados entre agosto de 2012 e janeiro de 2013, que preencheram os critérios de inclusão: (1) internação hospitalar psiquiátrica, (2) assinatura do TCLE e (3) idade entre 18 e 65 anos. Pacientes com diagnóstico de *delirium* foram excluídos. Utilizou-se um formulário de pesquisa, a Escala Breve de Avaliação Psiquiátrica e a Escala de Agressividade Declarada (OAS). O estudo foi aprovado pelo Comitê de Ética em Pesquisa da UFSM. A amostra foi composta por 137 pacientes. A prevalência de agressividade nas primeiras 24 horas foi de 41,6%; de agressividade verbal, 37,2%; agressividade física, 8,8%; contra objetos, 8,8% e autoa-gressão, 5,5%. Os principais fatores de risco foram o caráter involuntário da internação, história de qualquer tipo de agressão na semana anterior, história de uso compulsivo de maconha e cocaína, hipótese diagnóstica de transtorno de uso de múltiplas substâncias e tabagismo. Os pacientes agressivos de maneira geral exibiram maior pontuação geral na escala BPRS e nos componentes ativação, distúrbios do pensamento e retraimento-retardo, com menor pontuação no componente ansiedade-depressão. Os sujeitos agressivos fisicamente apresentaram agitação e comportamento psicótico mais graves. Os autoagressivos, além disso, exibiram maior desorientação e desorganização do pensamento, sendo considerados os pacientes mais graves do estudo. Concluiu-se que a agressividade nas primeiras 24 horas de internação está relacionada com a história de comportamento agressivo na semana anterior, história de uso de substâncias e, principalmente, com a gravidade da psicopatologia, sendo mais grave a agressividade quanto mais grave for a psicose e a agitação do paciente. Pacientes muito agitados e com graves sintomas psicóticos devem ser observados com cuidado especial pelo risco iminente de agressão física a outros e autoagressão.

### ANÁLISE DA EXPRESSÃO DE GENES RECEPTORES E REGULADORES DE NEUROTRANSMISSORES EM UM NOVO MODELO ANIMAL PARA ESQUIZOFRENIA

MARCOS LEITE SANTORO, CAMILA MAURICIO SANTOS, VANESSA K. OTA, MARIANA C. DIANA, LETICIA SPINDOLA, PATRICIA NATALIA SILVA, ARY GADELHA, RODRIGO A. BRESSAN, VANESSA C. ABILIO, SINTIA IOLE BELANGERO

UNIVERSIDADE FEDERAL DE SÃO PAULO - UNIFESP

Recentemente, nosso grupo mostrou que a linhagem Spontaneously Hypertensive Rats (SHR) apresentava uma série de comportamentos, classicamente, associados com modelos animais de esquizofrenia. O presente projeto propõe investigar se há diferença na expressão de genes de receptores e reguladores de neurotransmissores no tecido sanguíneo e duas regiões cerebrais (nucleus accumbens - NAcc e córtex pré-frontal - CPF) entre a linhagem de ratos SHR (N=8), modelo de esquizofrenia, e a linhagem de ratos Wistar, linhagem controle, (N=8), ambos tratados com veículo, e entre os grupos SHR tratado e não tratado com antipsicóticos (risperidona - N=8, clozapina - N=7, haloperidol - N=7). Nós utilizamos a técnica de PCRarray para verificar a expressão de 84 genes relacionados a neurotransmissão. Na comparação entre as linhagens SHR e Wistar encontramos quatro genes diferencialmente expressos no CPF (Chrnb4, Gad2, Qrfpr e Slc5a7) e um no sangue (Sstr4). Além disso, observamos que o gene Tacr3 apresenta uma expressão diminuída no CPF e aumentada no NAcc do SHR em relação ao Wistar e que esse gene está correlacionado positivamente entre o sangue e NAcc e negativamente entre o sangue e CPF dos animais SHR. Com relação aos grupos tratados com antipsicótico, encontramos três genes diferencialmente expressos no CPF do grupo clozapina (Drd2, Drd3 e Brs3) e dois no CPF do grupo haloperidol (Brs3 e Sstr4). Nosso trabalho apontou genes diferencialmente expressos entre as duas linhagens que estão envolvidos em importantes vias relacionadas à entre os grupos SHR não tratado e tratado com antipsicóticos apontou genes, possivelmente, relacionados ao efeito do antagonismo de receptores D2-like e associados à melhora no comportamento, como observado por nós anteriormente. Estes resultados demonstram semelhanças genéticas com a esquizofrenia nessa linhagem, além de apontar um potencial biomarcador e novos genes, possivelmente, envolvidos com o fenótipo do SHR e com resposta aos antipsicóticos.

### AVALIAÇÃO DE FATORES NEUROTRÓFICOS E MARCADORES IMUNOLÓGICOS EM INDIVÍDUOS COM TRANSTORNO BIPOLAR

IZABELA GUIMARÃES BARBOSA

UNIVERSIDADE FEDERAL DE MINAS GERAIS

O transtorno bipolar (TB) é uma síndrome psiquiátrica de prevalência elevada, associada a altas taxas de recorrência e suicídio. Evidências científicas recentes têm demonstrado o envolvimento dos fatores neurotróficos e marcadores imunológicos nas regulações de neurotransmissores, plasticidade sináptica, expressão gênica, sobrevivência e morte neuronal como os principais fatores associados à fisiopatologia do TB. As relações entre níveis circulantes de BDNF nos diversos transtornos neuropsiquiátricos e alterações imunológicas em pacientes com TB foram apresentadas nos artigos 1 e 2. O objetivo da tese foi avaliar a concentração plasmática dos níveis de fatores neurotróficos, parâmetros imunológicos (TNF-α, sTNFR1, sTNFR2, adiponectina, leptina, resistina, CCL2, 3, 11 e 24, e CXCL8 e 10), bem como a frequência leucocitária e ativação das vias intracelulares de pacientes com o diagnóstico de TB em comparação ao controle. Foram incluídos nesse estudo 93 pacientes com o diagnóstico de TB tipo I e 58 controles. Os sujeitos foram avaliados pelo Mini-Plus, escalas de mania de Young e de depressão de Hamilton. Também foi realizada avaliação de comorbidades clínicas, IMC, e avaliação cognitiva (incluindo o mini exame do estado mental e bateria de avaliação frontal). Níveis plasmáticos de BDNF, NGF, GDNF, TNF-α, sTNFR1, sTNFR2, adipocinas e quimiocinas foram avaliados por ELISA. A frequência de populações leucocitárias e de ativação das vias NF-κB (p65) e MAPKs (ERK1/2 e p38) foi feita por FACS e por Western blot. A caracterização demográfica, presença de comorbidades clínicas e psiquiátricas, presença de tentativas de suicídio, e dados clínicos referentes ao próprio TB foram realizados e encontram-se descritos no artigo 3. Os artigos 4, 5 e 6 analisaram os níveis plasmáticos de fatores neurotróficos em pacientes com TB em comparação a controles. No artigo 4 demonstramos que pacientes com TB de longa duraçâo apresentam elevação dos níveis de BDNF. No artigo 5 demonstramos que pacientes com TB e em mania apresentam redução dos níveis de NGF. No artigo 6 foi demonstrado que pacientes com o diagnóstico de TB apresentam aumento dos níveis de GDNF. Moléculas relacionadas ao sistema imunológico em pacientes com o diagnóstico de TB foram avaliados nos artigos 7, 8 e 9. No artigo 7 demonstramos que pacientes com TB apresentam aumento dos níveis de adiponectina, leptina e sTNFR1 em relação ao controle. No artigo 8 demonstramos que pacientes com TB apresentam elevações nos níveis de CCL11, CCL24, CXCL10 e redução dos níveis de CXCL8. No artigo 9 demonstramos que pacientes com TB apresentam deficit frontal, assim como as relações desta comorbidade com níveis de fatores neurotróficos e marcadores imunológicos. Pacientes com TB apresentam aumento de monócitos CD14+, redução de linfócitos T reguladores CD4+CD25+FOXp3+, e aumento da fosforilação de NF-κB/p65, ERK1/2 e p38, possíveis mecanismos intracelulares associados a fisiopatologia do TB. O presente estudo fortalece a visão de que o TB é associado a alterações em fatores neurotróficos e parâmetros imunológicos. 1. Teixeira AL, Barbosa IG, Diniz BS, et al. Circulating levels of brain-derived neurotrophic factor: correlation with mood, cognition and motor function. Biomark Med. 2010;4(6):871-87. 2. Barbosa IG, Huguet RB, Neves FS, et al.Imunologia do transtorno bipolar. J Bras Psiquiatr. 2009;58(1):52-9. 3. Barbosa IG, Ferreira SA, Huguet RB, et al. Comorbidades clínicas e psiquiátricas empacientes com transtorno bipolar do tipo I. J Bras Psiquiatr. 2011;60(4):271-6. 4. Barbosa IG, Rocha NP, Miranda AS, et al. Increased BDNF levels in long-term bipolar disorder patients. Rev Bras Psiquiatr. 2013;35(1):67-9. 5. Barbosa IG, Huguet RB, Neves FS, et al. Impaired nerve growth factor homeostasis in patients with bipolar disorder. World J Biol Psychiatry. 2011;12(3):228-32. 6. Barbosa IG, Huguet RB, Sousa LP, et al. Circulating levels of GDNF in bipolar disorder. Neurosci Lett. 2011;502(2):103-6. 7. Barbosa IG, Rocha NP, de Miranda AS, et al. Increased levels of adipokines in bipolar disorder. J Psychiatr Res. 2012;46(3):389-93. 8. Barbosa IG, Rocha NP, Bauer ME, et al. Chemokines in bipolar disorder: trait or state? Eur Arch Psychiatry Clin Neurosci. 2013;263(2):159-65. 9. Barbosa IG, Rocha NP, Huguet RB, Fet al. Executive dysfunction in euthymic bipolar disorder patients and its association with plasma bio-markers. J Affect Disord. 2012;137(1-3):151-5.

### AVALIAÇÃO PSICOPATOLÓGICA DE SINTOMAS DO TRANSTORNO BIPOLAR NA INFÂNCIA E ADOLESCÊNCIA

PEDRO MARIO PAN, GIOVANNI ABRAHÃO SALUM, ARY GADELHA, TAIS MORIYAMA, HUGO COGO-MOREIRA, ANA SOLEDADE GRAEFF-MARTINS, MARIA CONCEIÇÃO ROSARIO, GUILHERME POLANCZYK, ELISA BRIETZKE, LUIS AUGUSTO ROHDE, RODRIGO AFFONSECA BRESSAN

ESCOLA PAULISTA DE MEDICINA DA UNIVERSIDADE FEDERAL DE SÃO PAULO

*Introdução:* A fenomenologia do transtorno bipolar na infância e adolescência é um tópico de intenso debate para clínicos e pesquisadores. Não existem critérios diagnósticos específicos para essa população nas classificações atuais, e a diferenciação entre sintomas psicopatológicos do transtorno bipolar e comportamentos esperados para a idade pode ser difícil, principalmente quando são considerados os sintomas de mania. Apesar disso houve um aumento significativo na identificação de casos em amostras populacionais nos últimos anos. Assim, determinar a estrutura dimensional dos sintomas de mania em grandes amostras comunitárias e sua associação com história familiar de transtornos psiquiátricos é fundamental para a melhor caracterização do diagnóstico clínico e a diferenciação entre manifestações normais e patológicas.

*Objetivo:* O objetivo principal deste estudo foi investigar a distribuição de sintomas mania-*like* em uma amostra comunitária de crianças de 6 a 12 anos, através da análise de estruturas latentes de sintomas. A validade dos constructos latentes identificados foi avaliada através da associação com medidas de impacto psicossocial e história familiar de psicopatologia.

*Métodos:* A amostra inicial foi composta por 2512 crianças de 6 a 12 anos provenientes de escolas públicas de São Paulo e Porto Alegre. A partir do relato dos pais, 479 (19,1%) indivíduos responderam positivamente a pergunta de rastreio sobre episódios de elevação anormal do humor e completaram a seção detalhada que abordava 26 sintomas de mania. Análise Fatorial Confirmatória avaliou a aderência dos dados da amostra a uma estrutura de duas dimensões de sintomas previamente relatada: a dimensão *“undercontrol”* e a dimensão *“exuberant”.* Teoria de Resposta ao Item investigou a habilidade de discriminar gravidade e frequência de cada sintoma avaliado. Análise de Classes Latentes identificou grupos de indivíduos de acordo com a frequência reportada dos sintomas mania-like. Foram então avaliadas as associações entre os constructos latentes descritos e: (1) comorbidades psiquiátricas, (2) impacto psicossocial e (3) história familiar de psicopatologia.

*Resultados:* A estrutura latente dimensional de sintomas de mania foi avaliada, e as dimensões previamente encontradas *“undercontrol”* e *“exuberant”* foram confirmadas em nossa amostra. Através da Teoria de Resposta ao Item, foi identificado que os sintomas da dimensão “undercontrol” têm uma maior capacidade de discriminar as manifestações mais graves de sintomas de mania. Por outro lado, a dimensão *“exuberant”* foi capaz de discriminar a variância relacionada às manifestações menos graves. A Análise de Classes Latentes permitiu a identificação de um grupo de pacientes com alta frequência de sintomas de mania”. Este grupo foi composto por pacientes gravemente comprometidos, com níveis significativos de comorbidades psiquiátricas (tais como Depressão, Transtorno de Déficit de Atenção e Hiperatividade e Transtorno Desafiador de Oposição) e prejuízo funcional. O impacto dos sintomas de mania no funcionamento psicossocial dos indivíduos foi demonstrado, e a dimensão *“undercontrol”* mostrou-se uma preditora estatisticamente significante de impacto no funcionamento mesmo após o controle de potenciais confundidores, como a ocorrência de transtornos psiquiátricos comórbidos. A relação entre os constructos latentes de sintomas de mania e a história familiar de psicopatologia foi determinada pela primeira vez na literatura. A dimensão *“undercontrol”* foi associada a história familiar de transtornos de humor (depressão e mania) e tentativas de suicídio. Ainda, indivíduos da classe latente mais gravemente comprometida apresentaram uma prevalência elevada de história familiar de transtornos de humor, comparável a amostras clínicas de Transtorno Bipolar.

*Conclusões:* O presente estudo confirmou uma estrutura dimensional de sintomas de mania previamente proposta e demonstrou, pela primeira vez, sua associação com história familiar de transtornos de humor. Estudos longitudinais com populações em risco são necessários, pois podem determinar a validade dos contructos latentes propostos como preditores para a transição entre manifestações sub-clínicas e o diagnóstico clínico de Transtorno Bipolar.

